# Synthesis and Cytotoxicity of Silicon and Germanium Containing Pyridine Oxime O-Ethers

**DOI:** 10.1155/S1565363303000232

**Published:** 2003

**Authors:** Edgars Abele, Ramona Abele, Pavel Arsenyan, Irina Shestakova, Iveta Kanepe, Inga Antonenko, Juris Popelis, Edmunds Lukevics

**Affiliations:** Latvian Institute of Organic Synthesis 21 Aizkraukles Street Riga LV-1006 Latvia

## Abstract

Silicon and germanium containing pyridine aldoxime, ketoxime and amidoxime O-ethers have been prepared using phase transfer catalytic systems oxime alkyl halide solid KOH 18-crown-6 benzene and oxime alkyl halide solid K_2_CO_3_ or Cs_2_CO_3_ 18-crown-6 toluene. Cytotoxic activity of silicon and germanium containing pyridine oxime O-ethers was tested in vitro on two monolayer tumor cell lines: MG- 22A (mouse hepatoma) and HT-1080 (human fibrosarcoma). O-[3-Yriethylsilylpropyl]- and O-[3-(1-methyl- 1-silacyclopentyl)propyl] oximes of pyridine aldehydes and ketones exhibit high cytotoxicity. Presence of methyl group in the pyridine ring considerably decreased activity of amidoxime O-ethers. Oxime ethers containing two elements are essentially inactive. For 2-acetylpyridine oxime ethers the activity increases in order of alkyl substituents: Et_3_GeCH_2_CH_2_SiMe_2_CH_2_ < Et_3_SiCH_2_CH_2_CH_2_ < (CH_2_)_4_SiCH_2_CH_2_CH_2_. Cytotoxicity of ketoxime O-ethers is considerably lower in comparison with aldoxime O-ethers.


					Synthesis and Cytotoxicity of Silicon and Germanium
Containing Pyridine Oxime O-Ethers
Edgars Abele*, Ramona Abele, Pavel Arsenyan, Irina Shestakova, Iveta Kanepe, Inga Antonenko,
Juris Popelis, and Edmunds Lukevics'
Latvian Institute ofOrganic Synthesis,
21 Aizkraukles Street, Riga, LV-IO06, Latvia; Fax." (371) 7550338," E-mail." abele@osi.lv
ABSTRACT
Silicon and germanium containing pyridine aldoxime, ketoxime and amidoxime O-ethers have been
prepared using phase transfer catalytic systems oxime alkyl halide solid KOH 18-crown-6 benzene and
oxime alkyl halide solid K2CO3 or Cs2CO3 18-crown-6 toluene. Cytotoxic activity of silicon and
germanium containing pyridine oxime O-ethers was tested in vitro on two monolayer tumor cell lines: MG-
22A (mouse hepatoma) and HT-1080 (human fibrosarcoma). O-[3-Yriethylsilylpropyl]- and O-[3-(1-methyl-
1-silacyclopentyl)propyl] oximes of pyridine aldehydes and ketones exhibit high cytotoxicity. Presence of
methyl group in the pyridine ring considerably decreased activity of amidoxime O-ethers. Oxime ethers
containing two elements are essentially inactive. For 2-acetylpyridine oxime ethers the activity increases in
order of alkyl substituents: Et3GeCHzCI-|2SiMezCH2 < Et3SiCHzCHzCH2 < (CH2)4SiCH2CHzCH2.
Cytotoxicity ofketoxime O-ethers is considerably lower in comparison with aldoxime O-ethers.
INTRODUCTION
Pyridine oxime O-ethers containing different heteroatomic groups are of interest as biologically active
compounds. For example, pyridine oxime amino derivatives exhibit antiandrogenic/1,2/, antibacterial/3/,
antidotal against organophosphate poisoning/4,5/, fungicidal and pesticidal /6/ activities. Sulfur derivatives
of pyridine oxime O-ethers display vasodilating, antiulcer /7/, trombocytes aggregation inhibiting /8/,
antibacterial /9/, cytotoxic/9/, antidotal of organophosphate poisoning/10/, fungicidal /11/and herbicidal
/12, 13/ activities. Phosphorus containing oxime ethers are reactivators of acetylcholinesterase /14/ and
exhibit insecticidal /15/ activity. Fungicidal and insecticidal properties of pyridine aldehyde O-
Dr. Hab. Chem. Edgars Abele and Prof. Edmunds Lukevics; Latvian Institute of Organic Synthesis, 21
Aizkraukles Street, Riga, LV-1006, Latvia; Tel: (371) 7551822, Fax: (37l) 7550338; E-mail: abele@osi.lv
299
Vol. 1, Nos. 3-4, 2003 Synthesis and Cytotoxicity ofSilicon and Germanium Containing
Pyridine Oxime O-Ethers
(triethylstannyl)oximes are also described/16/. Cytotoxicity of silicon and germanium derivatives of pyridine
oxime O-ethers has not been investigated till now and is the aim ofthe present work.
MATERIALS AND METHODS
Chemistry
IH NMR spectra were recorded on a Varian 200 Mercury instrument using CDCI3 as solvent and
hexamethyldisiloxane (HMDSO) as internal standard. Mass spectra were registered on a GC-MS HI?' 6890
(70 eV). GC analysis was performed on a Chrom-5 instrument equipped with flame-ionization detector using
glass column packed with 5 % OV-101 Chromosorb W-HP (80-100 mesh) (1.2 m x 3 mm). Pyridine oximes
1-3 and 13-16 were obtained according to the procedures described in /17, 18/. 1-(3-lodopropyl)-l-
methylsilacyclopentane was obtained by Grignard reaction /19, 20/. MeEt2SiCH2CH2Si(Me2)CH2I and
Et3GeCH2CHSi(Me2)CHI were prepared as described in reference /21/. The purity of all synthesized
compounds was determined by HPLC and content of impurities was not higher than 2.5%.
General procedure for alkylation of pyridine aldoximes and ketoximes 1-3.
Haloalkylsilane (1 mmol) was added to a suspension of pyridine oxime 1-3 (1 mmol), 18-crown-6 (0.026
g, 0.1 mmol) and powdered KOH (0.168 g, 3 mmol) in benzene (1 ml). The reaction mixture was stirred for
3-6 h at reflux (GLC control). The resulting mixture was filtered, benzene was removed under reduced
pressure and the residue was purified by column chromatography (benzene-ethyl acetate in different ratio
was used as eluent) to give products 4-12. The results can be seen in Tables 1, 3 and 4.
General procedure for alkylation of pyridine amidoximes 13-16
Haloaikylsilane (1 mmol) was added to a suspension of pyridine amidoxime 13-16 (1 mmol), 18-crown-6
(0.026 g, 0.1 mmol) and powdered K2CO3 (0.41g, 3 mmol) (in the synthesis of compounds 19,20 CszCO3
was used) in toluene (2 ml). The reaction mixture was stirred for 6-12 h at reflux (GLC control). The
resulting mixture was filtered, benzene was removed under reduced pressure and the residue was purified by
column chromatography (benzene-ethyl acetate in different ratio was used at eluent) to give products 17-25.
The results can be seen in Tables 2-4.
In vitro cytotoxicity assay
Monolayer cell lines were cultivated for 72 h in DMEM standard medium without an indicator and
antibiotics. After the ampoule was unfrozen not more than four passages were performed. The control cells
and cells with tested substances in the range of (2-5) x 104 cell/mL concentration (depending on line nature)
were placed on separate 96 wells plates. Solutions containing test compounds were diluted and added in
300
Edgars Abele et al. Bioinorganic ChemisttT andApplications
wells to give the final concentrations of 50, 25, 12.5, and 6.25 lag/mL Control cells were treated in the same
manner only in the absence of test compounds. Plates were cultivated for 72 h. The amount of surviving cells
was determined using crystal violet (CV) or 3-(4,5-dimethylthiazol-2-.yl)-2,5-diphenyltetrazolinium bromide
(MTT) coloration, which was assayed by multiscan spectrophotometer. The quantity of live cells on control
plate was taken in calculations for 100%/22, 23/. Concentration ofNO was determined according to/22/.
RESULTS AND DISCUSSION
Chemistry
Alkylation of pyridine oximes with silicon and germanium containing alkyl halides was studied. The
known methods for the preparation of oxime O-ethers are based on oxime alkylation in the presence ofNail
DMF/24/or alkali metal alkoxide/25, 26/. The oxime alkylation reactions in the phase transfer catalytic
(PTC) systems oxime RX (X Br, I)./ 10 % aqueous NaOH R4NX (R n-Bu, n-Oct; X Br, HSO4)
Phil/27, 28/, oxime / RX (X Br, I) /solid K/CO3 / 18-crown-6 / Phil/29/, oxime / RC1 / solid KOH or
KzCO3 solid KI 18-crown-6 PhMe or Phil/30/and oxime O-acetate or benzoate RX (X Br, I) solid
KOH / 18-crown-6 / Phil/31/considerably simplifies the preparation oftheir O-ethers.
The PTC system iodo- or bromomethylsilane solid KOH 18-crown-6 benzene was found to be the
most active in the synthesis of oxime ethers 4 12 from pyridine aldoximes and ketoximes 3 (Table 1, 3,
4). Synthesis of all pyridine oxime O-ethers proceeded stereoselectively with formation of only E-isomers
(yields up to 65%).
R'R"R'"Si(CH2)n X solid KOH
18-crown-6 Phil reflux
1 3 4-12
n SiR'R"R'"
However, the attempts to alkylate the pyridine amidoximes 13-16 with silicon containing alkyl halides in
PTC system (solid KOH / 18-crown-6 / benzene) were uneffective. Replacement of the solid KOH / 18-
crown-6 benzene to solid K2CO3 (or Cs2CO3) 18-crown-6 toluene increased the yield of pyridine
amidoxime O-ethers 17-25 (Tables 2-4). The E-isomers of products 17-25 were isolated by column
chromatography in 40-79 % yields.
R
NN N
O
H
H
13-16
R'R"R'"Si(CH2)n X solid K2CO or Cs2CO3
-NO(CH2)
R
18-crown-6 Phil reflux
N NH2
17-25
n SiR'R"R'"
301
Vol. 1, Nos. 3-4, 2003
Oxime
Synthesis and Cytotoxicity ofSilicon and Germanium Containing
Pyridine Oxinte O-Ethers
Table 1
Synthesis of pyridine aldoxime and ketoxime O-ethers 4-12
Pyridyl
1 2-
1 2-
1 2-
2 2-
2 2-
2 2-
2 2-
3 4-
3 4-
R SiR'R"R'" n
H
H
H
Me
Me
Me
Me
H
H
Yields of isolated products
Oxime
S!(Me2)CH2CH2GeEt3
SiEt. 3
1-methyl-1-silacyclopentyl 3
Si(Me2)CH2CH2SiEt2Me
Si(Me2)CH2CH2GeEt3
SiEt3 3
1-methyl-1-silacyclopentyl 3
SiEt3 3
1-methyl-l-silacyclopentyl 3
X
Br
Br
Br
Reaction
time, h
Product
Yield",
%
3 4 42
6 5 48
6 6 31
3 7 51
3 8 56
6 9 41
6 10 65
6 11 62
6 12 64
Table 2
Synthesis ofpyridine amidoxime O-ethers 17-25
Pyridyl R
13 3-
13 3-
14 4-
14 4-
15 2
15 2
15 2
16 2
16 2
Time,
SiR'R"R'" n X
h
H SiEts 3 Br 6
H 1-methyl-1-silacyclopentyl 3 7
H SiEt3 3 Br 12
H 1-methyl-1-silacyclopentyl 3 12
3-Me SiEt3 3 Br 8
3-Me 1-methyl-1-silacyclopentyl 3 6
3-Me Si(Me2)CH2CH2SiEtzMe 8
6-Me SiEt3 3 Br 7
6-Me Si(Me2)CH2CH2GeEt3 9
Yields of isolated products
Product
Yielda,
%
17 40
18 65
19 41)
20 33
21 4!;)
22 59
23 46
24 60
25 64
In Vitro cytotoxicity
Cytotoxic activity of silicon and germanium containing pyridine oxime O-ethers was tested in vitro on
two monolayer tumor cell lines: MG-22A (mouse hepatoma) and HT-1080 (human fibrosarcoma).
Concentrations providing 50% of tumor death effect were determined according to the known procedure/32/
using 96 well plates.
302
Edgars Abele et al. Biohorganic Chemisoy andApplications
Table 3
Mass spectroscopic data ofpyridine oxime O-ethers
Compound
10
11
12
17
18
19
2O
21
22
23
24
25
M/z (intensity, %)
337 (< 1, M+-C3H7), 219 (4), 193 (63), 163 (100), 149 (5), 133 (16), 119 (9), 103 (14), 92
(12), 78 (10), 59 (21).
279 (< 1, M+), 249 (23), 233 (8), 145 (7), 115 (21), 103 (100), 87 (41), 78 (74), 59 (30),
51 (14)
262 (2, M+), 217 (9), 203 (89), 192 (8), 184 (18), 175 (17), 165 10), 148 (40), 133 (13),
119 (18), 101 (84), 87 (19), 78 (100), 61 (23), 45 (37)
321 (< 1, M+-Me), 291 (3), 207 (51), 177 (100), 136 (16), 119 (13), 101 (14), 87 (15), 78
(45), 59 (26), 45 (26
367 (<1, M+-Et), 207 (73), 177 (100), 161 (5), 147 (5), 136 (17), 103 (130), 78 (47), 59
(16), 43 (7)
291 (< 1, M+), 247 (7), 133 (25), 119 (72), 87 (22), 78 (100), 59 (17), 51.(10)
276(< 1, M+), 231 (5), 133 (18), 119 (50), 99 (18), 78 (100), 45..(10)
249 (35, M+-Et), 115 (53), 103 (100), 87 (62), 75 (66), 59 (34)
262 (5, M+), 233 (5), 184 (41), 133 (5), 101 (100), 87 (13), 78 (23), 61 (13), 51 (18), 43
(1)
293 (2, M+), 264 (12), 222 (4), 137 (23), 120 (100), 103 (68), 87 (34), 78 (30), 59 (28),
51 (16)
276 (4, M+), 248 (12), 234 (14), 220 (13), 193 (8), 163 (10), 137 (20), 129 (9), 120 (100),
102 (66), 87 (27), 78 (66), 59 (24), 45 (46)
293 (2, M+), 264 (51), 222 (5), 137 (23), 120 (100), 103 (81), 87 (43), 78 (36), 59 (33),
43(10)
277 (6, M+), 248 (47), 234 (34), 220 (10), 193 (8), 178 (12), 148 (45), 137 (33), 129 (10),
120 (93), 102 (100), 87 (29), 78 (77), 59 (26), 51 (50), 43 (45)
307 (2, M+), 262 (6), 162 (13), 151 (10), 134 (100), 118 (16), 103 (18), 92 (36), 75 (13),
59(18)
290 (2, M+), 276 (6), 177 (16), 162 (22), 145 (10), 134 (100), 119 (26), 101 (20), 92 (66),
65 (29), 45 (22)
306 (2, M+-Et-Me), 222 (75), 192 (89), 134 (13), 119 (16), 101 (16), 92 (20), 74 (100), 59
(26), 45 (25)
307 (3, M+), 278 (7), 262 (10), 178 (10), 162 (41), 151 (24), 134 (100), 119 (32), 103
(34), 92 (66), 75 (22), 59 (35), 43(12)
396 (<1, M+-Me), 222 (100), 192 (46), 133 (14), 119 (28), 103 (14), 92 (14), 74 (97), 59
(17)
303
Vol. 1, .Nos. 3-4, 2003 Synthesis and Cytotoxicity ofSilicon and Germanium Containing
Pyridine Oxime O-Ethe
Table 4
1H NMR data ofpyridine oxime O-ethers
10
11
12
17
18
Ether ppm, CDCI3 HMDSO)
0.06 (s, 6H, Si(CH3)2), 0.67 (m, 10H, SiCH2 and GeCH2), 0.99 (t, 9H, J 7,6 Hz,
GeCH2CH3), 4.06 (s, 2H, OCH2), 7.23 (m, 1H, 5-H), 7.62- 7.79 (m, 2H, 3-H and 4-H),
8.13 (s, 1H, CH), 8.59 (m, 1H, 6-H)
0.52 (m, 8H, SIGH2), 0.92 (t, 9H, J 7.4 Hz, CH3), 1.60 (m, 2H, CHzC__HzCH2), 4.16 (t,
2H, J 7.0 Hz, OCH2), 7.26 (m, 1H, 5-H), 7.62 7.80 (m, 2H, 3-H and 4-H), 8.16 (s, H,
CH), 8.61 (m, 1H, 6-H)
0.08 (s, 3H, CH3), 0.59 (m, 6H, SiCH), 1.55 (m, 6H, CH(CH__z)2CH2 in silacycle and
CH2CH2CH2), 4.18 (t, 2H, J 7.0 Hz, OCH2), 7.25 (m, 1H, 5-H), 7.67 7.80 (m, 2H, 3-H
and 4-H), 8.16 (s, H, CH), 8.60 (m, H, 6-H)
-0.09 (s, 3H, SIGH3), 0.10 (s, 6H, Si(CH3)2), 0.51 (m, 8H, SiCH2), 0.91 (t, 6H, J 7,6 Hz,
SiCHzCH__3), 2.31 (s, 3H, CCH3), 4.06 (s, 2H, OCH), 7.36 (m, 1H, 5-H), 7.45 (m, 1H, 3-
H), 7.90 (m, 1H, 4-H), 8.59 (m, 1H, 6-H)
0.10 (s, 6H, Si(CH3)z), 0.72 (m, 10H, SiCH2 and GeCH), 0.99 (t, 9H, J 7.2 Hz,
GeCHzCH3), 2.29 (s, 3H, CCH3), 4.05 (s, 2H, OCH2), 7.21 (m, 1H, 5-H), 7.64 (m, H, 3-
H), 7.89 (m, 1H, 4-H), 8.58 (m, 1H, 6-H)
0.54 (m, 8H, SiCH2), 0.93 (t, 9H, J 7.6 Hz, CH3), 1.60 (m, 2H, CHzCH__zCH2), 2.35 (s,
3H, CH3), 4.17 (t, 2H, J 7.0 Hz, OCH2), 7.25 (m, 1H, 5-H), 7.61 (m, 1H, 3-H), 7.87 (m,
H, 4-H), 8.58 (m, H, 6-H)
0.09 (s, 3H, CH3), 0.60 (m, 6H, SiCH2), 1.55 (m, 6H, CH2(CH__z)2CH2 in silacycle and
CH2CH2CH2), 2.32 (s, 3H, CH3), 4.18 (t, 2H, J 7.0 Hz, OCH2), 7.25 (m, 1H, 5-H), 7.65
(m, H, 3-H), 7.89 (m, H, 4-H), 8.59 (ITI, H, 6-H)
0.54 (m, 8H, SiCH2), 0.93 (t, 9H, J 7.6 Hz, CH3), 1.65 (m, 2H, CH2CH__2CH2), 4.15 (t,
2H, J 7.0 Hz, OCHz), 7.42 (m, 2H, 3-H and 5-H), 8.01 (s, 1H, CH), 8.62 (m, 2H, 2-H
and 6-H)
0.09 (s, 3H, CH3), 0.59 (m, 6H, SiCHz), 1.55 (m, 6H, CHz(CHz)2CH2 in silacycle and
CH2CH2CH2), 4.16 (t, 2H, J 7.0 Hz, OCH2), 7.43 (m, 2H, 3-H and 5-H), 8.01 (s, 1H,
CH), 8.62 (m, 2H, 2-H and 6-H)
0.52 (m, 8H, SiCH2), 0.92 (t, 9H, J 7.0 Hz, CH3), 1.66 (m, 2H, CH2CH2CH2), 4.07 (t,
2H, J 7.0 Hz, OCH2), 4.77 (bs, 2H, NH2), 7.22 (m, 1H, 5-H), 7.91 (m, 1H, 4-H), 8.53 (m,
1H, 6-H), 8.85 (m, 1H, Z-H)
0.26 (s, 3H, CH3), 0.76 (m, 6H, SiCH2), 1.78 (m, 6H, CH2(CH2_)2CH2 in silacycle and
CH2CH2CH2), 4.21 (t, 2H, J 7.2 Hz, OCH2), 5.00 (bs, 2H, NH_), 7.48 (m, 1H, 5-H), 8.08
(m, 1H, 4-H), 8.77 (m, 1U, 6-H), 8.98 (m, 1H, 2-H)
304
Edgars Abele et al. Bioinorganic Chemistry andApplications
Table 4 (continued)
19
2O
21
22
23
24
25
0.52 (m, 8H, SiCHz), 0.93 (t, 9H, J 7.4 Hz, CH3), 1.65 (m, 2H, CH2CH2CH2), 4.06 (t,
2H, J 7.0 Hz, OCH2), 4.79 (bs, 2H, NH2), 7.51 (m, 2H, 3-H and 5-H), 8.62 (m, 2H, 2-H
and 6-H)
0.09 (s, 3H, CH3), 0.59 (m, 6H, SiCH2), 1.55 (m, 6H, CH(CH__z_)2CHg_ in silacycle and
CHzCHzCH2), 4.07 (t, 2H, J 6.8 Hz, OCHa), 4.79 (bs, 2H, NHa), 7.51 (m, 2H, 3-H and 5-
H), 8.63 (m, 2H, 2-H and 6-H)
0.52 (m, 81-t, SiCHz), 0.93 (t, 9H, J 6.8 Hz, CH), 1.72 (m, 2H, CHzCH2CH2), 2.56 (s,
3H, CH in ring), 4.03 (t, 2H, J 7.0 Hz, OCHz), 5.47 (bs, 2H, NHz),7.09 (m, 1H, 5-H),
7.45 (m, 1H, 4-H), 8.36 (m, 1H, 6-H)
0.08 (s, 3H, CH3), 0.60 (m, 6H, SiCHa), 1.55 (m, 6H, CHz(CH2)zCH: in silacycle and
CH2CI-IzCHa), 2.59 (s, 3H, CH in ring), 4.07 (t, 2H, J 6.8 Hz, OCHa), 5.50 (bs, 2H,
NHa), 7.17 (m, 1H, 5-H), 7.53 (m, 1.H, 4-H), 8.40 (m, 1H, 6-H)
-0.15 (s, 3H, EtzSiCH), 0.04 (s, 6H, Si(CH3)z), 0.43 (m, 8H, SiCHa), 0.82 (t, 6H, J 7.6
Hz, SiCHaCH3), 2.54 (s, 3H, CH in ring), 3.90 (s, 2H, OCH), 5.42 (bs, 2H, NHa), 7.10
(m, 1H, 5-H), 7.45 (m, 1H, 4-.H), 8.34 (m, 1H, 6-H)
0.53 (m, 8H, SiCH2), 0.90 (t, 9H, J 6.8 Hz, CH), 1.71 (m, 2H, CH2CHg_CHa), 2.51 (s,
3H, CH in ring), 4.05 (t, 2H, J 7.0 Hz, OCHz), 5.53 (bs, 2H, NHz),7.04 (m, IH, 5-H),
7.56 (m, 2H, 4-H and 3-H)
0.17 (s, 6H, Si(CH)2), 0.66 (m, 10H, SiCHa and GeCHz), 1.02 (t, 9H, J 7,0 Hz,
GeCHzCH), 2.63 (s, 3H, CH3 in ring), 4.05 (s, 2H, OCH), 5.60 (bs, 2H, NH2), 7.19 (m,
1H, 5-H), 7.68 (m, 2H, 4-H and 3-H)
The experimental evaluation of cytotoxicity is presented in Table 5. A preliminary analysis of the
structure-activity relationship for the cytotoxic action clearly indicates the strong influence of the silicon and
germanium containing alkyl substituent on toxic effects in vitro. In the series of O-silyl(germyl)alkyl
pyridine aldoximes and ketoximes (4-12) the TDs0 values change from 1.85 pg/mL (silacyclopentyl
derivative 6 on human fibrosarcoma HT- 1080 cell line) to non cytotoxic compounds (>100 lag/mL for 4, 7,
and 8). Oxime ethers containing two elements are essentially inactive. Silacyclopentyl derivative 6 exhibits
the higher cytotoxicity on HT- 1080 as compared to triethylsilylpropyl substituted pyridine aldoxime 5. For
pyridine ketoxime ethers (8-10) the activity increases in order of alkyl substituents: EtGeCHzCH_SiMe_CHa
< EhSiCHzCHzCHz < (CHz)4SiCI-IzCHzCH2. Cytotoxicity of ketoxime O-ethers is considerably lower in
comparison with aldoxime O-ethers.
In the cases of O-ethers of pyridine amidoximes the highest cytotoxicity was exhibited by unsubstituted
pyridine derivatives (17-20). The presence of methyl group in the pyridine ring considerably decreased the
activity of amidoxime O-ethers.
305
Vol. I, Nos. 3-4, 2003 Synthesis and Cytotaricity ofSilicon and Germanium Containing
Pyridine Oxime O-Ethers
Table 5
In vitro cell cytotoxicity and the ability of intracellular NO-generation by pyridine oxime O-ethers.
N HT -1080 MG -22A
TDso NO, o CVb
TDs0" NO, o CVb
4 >100 3 >100 6
5 3.5 350 5 350
6 1.85 400 6 500
7 >100 3 >100 4
8 >100 8 >100 4
9 62 350 29 500
10 28 175 27.5 300
11 3.65 350 3.5 350
12 3.15 300 5.5 200
17 5.4 .300 4.3 300
18 4.85 250 24.5 200
19 2.5 550 4.5 330
20 6 300 4.5 300
21 21.5 300 37 350
22 >100 9 >100 9
23 6.35 250 22 450
24 >100 4 >100 6
25 >100 4 >100 5
Concentration (g/mL) providing 50% cell killing effect [(CV+MTT)/2)
NO Concentration (%) (CV" coloration)
The NO level was determined accordingly/22/, NO release was defined using the Greyss reagent (by NO2
concentration in the cultural medium). The yield of nitrite was expressed as NO2 nmol/200 gL of cultural
medium in testing plates for 100% alive cells after CV coloration assay (pyridine derivatives concentration
100 lag/mL). It was shown (Table 5) that compounds 6, 9, and 19 readily increase NO concentration in the
cultural medium (TG00=500%) in HT-1080 and MG 22A cell lines.
306
Edgars Abele et al. Bioinorganic Chemistry andApplications
REFERENCES
1. Scherico Ltd. Brit. Pat. 1070964 (1967); Chem. Abstr., 68,104994x (1968).
2. F.J. Villant, US Pat. 3290320 (1966); Chem. Abstr., 66, 46340b (1967).
3. C.Y. Hong, Y.K. Kim, S.H. Kim, J.H. Chang, H. Chou, D.H. Nam, A.R. Kim, J.H. Lee and K.S. Park,
US Pat. 5776944 (1998); Chem. Abstr.,129, 122580s (1998).
4. T. Nishimura, C. Yamazaki and T. Ishiura, Bull Chem. Soc. Jap., 40, 2434 (1967).
5. I. Bulete, Rom. Pat. 51935 (1970); Chem. Abstr., 73, 120678f(1970).
6. F. Rohi, V. Harries, E. Ammermann, G. Lorenz, S. Strathmann, A. Ptock, H. Sauter, W. Grammenos, T.
Grote, H. Bayer, R. Kirstgen, K. Oberdorf, B. Muller and R.Muller, PCTInt. WO Pat. 9812179 (1998);
Chem. Abstr., 128, 257334q (1998).
7. C. Malen, B. Danree and X.Pascaud, Get. Pat. 2217180 (1972); Chem. Abstr., 78, 16052q (1973).
8. U. Niewoehner, U.E. Mueller, E. Perzbom, E. Bischoff and H.G. Dellweg, Eur. Pat. 471259 (1992);
Chem. Abstr., 116, 214360e (1992).
9. F. Trecourt, B. Gervais, O. Mongin, C. Le Gal, F. Mongin and G. Queguiner, J. Org. Chem., 63, 2892
(1998).
10. F. Hauschild, R. Schmiedel and W.D. Wiezorek, East. Ger. Pat. 38036 (1965); Chem. Abstr., 63,
13225c (1965).
11. C. Fest, W. Brandes, G. Haenssler and P. Reinecke, Get. Pat. 3608383 (1987); Chem. Abstro, 107,
217494b (1987).
12. W. Fuehrer, J. Stetter, H. Foerster, L. Eue.and R.R. Schmidt, Ger. Pat. 3221215 (1983); Chem. Abstr.,
100, 138961d (1984).
13. T. Goshima, Y. Kitagawa, S. Kaji, H. Hayakawa and A. Watanabe, Jpn. Pat. 0499767 (1992); Chem
Abstr., 117, 131079q (1992).
14. R.A. Andersen, J.A.B. Barstad and K. Laake, Progr. Brain Res., 36, 189 (1972).
15. lshikara Sangyo Kaisha, Ltd. Jpn. Pat. 8183495 (1981); Chem. Abstr., 95, 132689c (1981).
16. Farbenfabriken Bayer A.-G. Brit. Pat. 945068 (1963); Chem. Abstr., 60, 12051c (1964).
17. S. Ginsburg and I.B. Wilson, J. Am. Chem. Soc., 79, 481 (1957).
18. E. Bernasek, J. Org. Chem., 22, 1263 (1957).
19. N.S. Nametkin, K.S. Vdovin, K.S. Pushchevaya, V.I. Zavyalov, Izv. Akad. Nauk SSSR, Otd. Khim.
Nauk, 1453 (1965).
20. J.W. Wilt and C.F. Dockus, J. Amer. Chem. Soc., 92, 5813 (1970).
21. K. Rubina, E. Abele, P. Arsenyan, R. Abele, M. Veveris and E. Lukevics, Metal-Based Drugs, 8, 85
(2001).
22. D.J. Fast, R.C. Lynch and R.W. Leu, J. Leuckocyt. Biol., 52, 255 (1992).
23. P.J. Freshney, Culture ofAnimal Cells (A Manual ofBasic Technique), Wiley-Liss, New York, 1994,
pp. 296-297.
24. Z. Wimmer, D. Saman, J. Smolinkova and M. Romanuk, Lieb. Ann. Chem., 1091 (1988).
25. I. Hadegorn, W.-H. Gundel, J. Hosse and Chr. Fenter, Arzneim.-Forsch. (Drug Res.)., 26, 1273 (1976).
307
Vol. I, Nos. 3-4, 2003 Synthesis and Cytotoxicity ofSilicon and Germanium Containing
Pyridine Oxime O-Ethers
26. A. Steinhards and W. Mathes, US. Pat. 2924604 (1958); Chem. Abstr., 56, 736d (1961).
27. K. Rubina, Yu. Goldberg, A. Gaukhman and M. Shymanska, Synth. Commun., 19, 3129 (1989).
28. K. Rubina, Yu. Popelis, Yu. Goldberg and M. Shymanska, Chem.. Heterocycl. Comp., 28, 1406 (1992).
29. E. Abele, Yu. Popelis, E. Lukevics, M. Shymanska and Yu. Goldberg, Chem. Heterocycl. Comp., 30, 14
(1994).
30. E. Abele, R. Abele, K. Rubina, J. Popelis, I. Sleik,a and E. Lukevics, Synth. Commun., 28, 2621
(1998).
31. E./bele, R. bele, J. Popelis and E. Lukevics, Latv. J. Chem., N 2, 61 (1998).
32. R.J. Riddell, R.H. Clothier, M: Fd.Balls, Chem. Toxicol., 24, 469 (1986).
3O8

				

